# Laser-induced inactivation of *Plasmodium falciparum*

**DOI:** 10.1186/1475-2875-11-267

**Published:** 2012-08-08

**Authors:** Danielle LeBlanc, Robert Story, Eitan Gross

**Affiliations:** 1 Department of Physics, University of Arkansas, Fayetteville, AR, 72701, USA; 2 Biomass Research Center, University of Arkansas, Fayetteville, AR, 72701, USA

**Keywords:** Dialysis, Escherichia coli, Haemozoin, Plasmodium falciparum, Pulsed laser, Third harmonic generation

## Abstract

**Background:**

Haemozoin crystals, produced by *Plasmodium* during its intra-erythrocytic asexual reproduction cycle, can generate UV light via the laser-induced, non-linear optical process of third harmonic generation (THG). In the current study the feasibility of using haemozoin, constitutively stored in the parasite’s food vacuole, to kill the parasite by irradiation with a near IR laser was evaluated.

**Methods:**

Cultured *Plasmodium* parasites at different stages of development were irradiated with a pulsed NIR laser and the viability of parasites at each stage was evaluated from their corresponding growth curves using the continuous culture method. Additional testing for germicidal effects of haemozoin and NIR laser was performed by adding synthetic haemozoin crystals to *Escherichia coli* in suspension. Cell suspensions were then irradiated with the laser and small aliquots taken and spread on agar plates containing selective agents to determine cell viability (CFU).

**Results:**

Parasites in the late-trophozoites form as well as trophozoites in early-stage of DNA synthesis were found to be the most sensitive to the treatment with ~4-log reduction in viability after six passes through the laser beam; followed by parasites in ring phase (~2-log reduction). A ~1-log reduction in *E. coli* viability was obtained following a 60 min irradiation regimen of the bacteria in the presence of 1 μM synthetic haemozoin and a ~2-log reduction in the presence of 10 μM haemozoin. Minimal (≤15%) cell kill was observed in the presence of 10 μM haemin.

**Conclusions:**

Laser-induced third-harmonic generation by haemozoin can be used to inactivate *Plasmodium*. This result may have clinical implications for treating severe malaria symptoms by irradiating the patient’s blood through the skin or through dialysis tubing with a NIR laser.

## Background

Malaria is a devastating disease killing more than 800,000 people a year worldwide [1]. Malaria is caused by the parasite *Plasmodium* vectored by mosquitoes. The parasite infects erythrocytes where it replicates [2]. The development of human vaccines is hampered by a complex intra-erythrocytic eukaryote pathogen and lack of a persistent memory immune response to malaria. Due to the chronic nature of some *Plasmodium* strains, both T cells and B cells become less functional. Furthermore, *Plasmodium* has several life stages, making selection of important antigens for targeting in a vaccine more challenging [3]. Several classes of drugs are currently in use to treat malaria. These include quinolines, antifolates, and artemisinin-combination therapy (ACT). Quinolines are haemozoin inhibitors which bind to purified haem and associate with haemozoin-containing fractions from *Plasmodium*, inhibiting the conversion of haem to haemozoin [4]. Antifolates block folic acid synthesis, which is essential to *Plasmodium* growth because the parasite is unable to utilize pyrimidines already synthesized by the host and must use this pathway to make its own. Artemisinins are activated by haem or free iron to generate parasiticidal radicals. Unfortunately, the long-term efficacy of the quinolines and antifolates has been limited due to the fast emergence of drug-resistant *Plasmodium* strains [5].

To overcome these hurdles, haemozoin, naturally present within the parasite, is being proposed here to be used as a “localized” source of UV radiation to kill the parasite. Since the proposed treatment requires illuminating the parasite with a laser, the technique can only be used at present to kill the parasite in the blood circulation but not the liver or other deep-tissue organs. Haemozoin is produced by *Plasmodium* during its intra-erythrocytic asexual reproduction cycle [6]. The digestion of hemoglobin releases monomeric alpha-haematin (ferriprotoporphyrin IX). This compound is toxic, since it is a pro-oxidant and catalyzes the production of reactive oxygen species. *Plasmodium*, therefore, detoxifies the haematin, by bio crystallization—converting it into insoluble and chemically-inert beta-haematin crystals (called haemozoin). In *Plasmodium* the food vacuole fills with haemozoin crystals, which have dimensions of 100x100x300 nanometers and each contain about 80,000 haem molecules [7].

Haemozoin exhibits a very strong third harmonic generation (THG) signal [8]. In THG, a compound converts three photons of the laser light within the focus of a laser beam into one emitted photon of triple the frequency. This frequency conversion phenomenon strictly depends on the physical property of third-order dielectric susceptibility of the material (a more detailed account of THG is given in Additional file 1). Since the frequency of a photon (ν) is inversely related to its wavelength (λ) according to the equation ν ~ C/λ (where C is the speed of light), the emitted photon will have a wavelength which is a third of the wavelength of the fundamental laser wavelength. Furthermore, using a Taylor series approximation, it can be shown that the intensity of the THG signal scales as the third power of the incident laser light intensity (see Additional file 1). A schematic illustrating third harmonic generation using a black-box diagram and energy levels is shown in Additional file 2.

UV light has been shown to offer an effective germicidal treatment against a broad range of pathogens including viruses [9], bacteria [10], fungi [11] and protozoa [12]. Thus, it may be plausible to treat infected individuals with a transcutaneous NIR laser; or by attaching the patient to a dialysis machine and passing the blood through narrow tubing equipped with a NIR-transparent window through which the blood can be irradiated. To generate THG at the germicidal wavelength of 265 nm, the blood needs to be irradiated with NIR light at 795 nm (795 = 3 x 265 nm). Light at 795 nm is relatively harmless to the patient thanks to a dip in oxyhaemoglobin absorption spectrum [13]. Since humans do not produce haemozoin the co-lateral damage to the host’s cells should in principle be low and the therapeutic ratio high.

The results of feasibility experiments carried out with cultures of erythrocytic *Plasmodium falciparum* are being reported here. The data suggest that a ~0.5-log reduction in parasite count can be achieved by passing the entire volume of the blood sample once through the path of a single laser beam with an intensity of ~0.5 W/cm^2^. At a standard perfusion rate of 300 ml/min it would take ~20 minutes to circulate the entire volume of blood of an adult male patient (~6 liters) through the dialysis machine to achieved a ~0.5-log reduction in parasitaemia. Treatment length can be shortened by adding more lasers along the dialysis tubing line.

## Methods

### Chemicals

Haemin was obtained from Sigma (St. Louis) and 10 mg was dissolved in 1 ml of DMSO to which 99 ml of 6 M sodium acetate pH 4.8 was added. The solution was heated to 60 ^o^C with constant magnet stirring for 16 - 17 hours. The suspension was centrifuged at 12,000 g for 15 min after addition of 100 μl of 10% SDS. The suspension was washed multiple times in 2% SDS and 100 mM sodium bicarbonate until supernatant was clear, then washed five times with 2% SDS and ten times in distilled water. The suspension of haem crystals was quantitated after decrystallization in 20 mM sodium hydroxide [7].

### Plasmodium protocol

*Plasmodium falciparum* HB-3 (ATTC 50113) from a frozen vial was placed in culture and maintained by the continuous flow technique [14]. For experiments, cultures were initiated with a 10% suspension of a human A + erythrocytes in RPMI 1640 medium containing 10% human A + serum at a starting parasitaemia of 0.2% as described by Waki *et al*[15]. Cultures were incubated in a cell culture incubator at 37°C with a gas mixture containing 5% CO_2_, 10% O_2_ and 85% N_2_. Triplicate cultures (0.5 ml) were prepared in 24-well flat-bottom tissue-culture plates and multiplication of parasites monitored daily using Giemsa-stained thin films made from each of the cultures. For determination of growth ~10,000 erythrocytes were examined at 1,000x magnification under oil.

Two methods were used to synchronize parasites as previously described [15]. First, cells were treated three times with D-sorbitol at 0, 48 and 88 hours. The 88 hr treatment selects for a relatively narrow age distribution of newly formed rings (“fine tuning”) [16]. Prior to transition from schizont to ring form the parasites were treated again with sorbitol to obtain young ring form. In the second method, parasites in the stage of DNA synthesis were removed from the culture by treating the cells with 50 mM hydroxyurea for six hours. Parasites in trophozoite stage were prepared by cultivating the young ring form parasite for 18 hours or 30 hours. The 18-hr trophozoites which had just transformed from ring forms and those that remained as trophozoites after 30 hours in culture were hydroxyurea-sensitive and were designated early (ES) and synthesis (S) phases, respectively [15].

For irradiation, 60 ml infected blood cells were loaded onto a sterile reservoir and passed multiple times through a quartz flow cell cuvette with a 6.5 mm wide x 6.5 mm high aperture and a 5 mm path length (Starna, Atascadero, CA) at a flow rate of 1.0 ml/sec using a peristaltic pump with sterile tubing. Cells were irradiated with the laser from a distance of 10 cm. Following irradiation a 1-ml aliquot was diluted with a 10% fresh erythrocyte suspension to provide un-irradiated host cells for the parasite and was put into culture dish and return to standard culture conditions. The number of pRBC was monitored daily and the results plotted as the ratio of the initial number.

The time it took to reduce pRBC count to 37% of its initial value is referred to as the time constant (*τ*) for parasite inactivation. Tao (*τ*) was calculated from the slope of the kill curves (the plots of Log(N_o_/N) vs. time) by assuming a first-order kill reaction kinetics (N = N_o_e^-t/τ^). The corresponding energy (*E*_*o*_) of NIR light needed to reduce parasite count to 37% of its initial value was calculated using the relation *E*_*o*_ *= Pτ*, were *P* is the laser output power at 800 nm. Experimental values for both *E*_*o*_ and *τ* are listed in Table 1.

**Table 1:** Dose-response data for NIR-laser germicidal effect

**Organism**	τ, sec*	**E**_**o**_**, J/cm**^**2#**^
*Plasmodium falciparum*		
Late trophozoites (S)	34.8±3.9	16.6±1.7
Early trophozoites (ES)	35.6±3.6	17.2±1.7
Ring	60.2±6.4	29.1±3.0
*Escherichia coli*		
+10 μM haemozoin	711±8.1	345.2±40
+1.0 μM haemozoin	(165±14)·10^1^	800±8.4
+10 μM haemin	(104±11)·10^3^	(504±51)·10^2^

^*^τ is the time to reduce parasite count to 37% of its initial value following irradiation with the laser.

^#^*E*_*o*_ is the energy of NIR light needed to reduce parasite count to 37% of its initial value.

### *Escherichia coli* protocol

*Escherichia coli* (*E. coli,* ATCC 11775) from an agar slant were inoculated into 6 ml nutrient broth (Becton Dickinson/Difco) and incubated at 37°C in a cell culture incubator. After 18 h incubation, cells (~1 ·10^8^ CFU/ml) were diluted 10^6^-fold into BHI (Becton Dickinson/Difco) broth and placed in a stirred quartz cuvette containing haemozoin or haemin for irradiation. Cuvettes containing 3 ml cell suspension were placed in a cell holder and irradiated with the laser for various time periods at room temperature. Following irradiation cell samples (0.1 ml) were spread onto agar plates containing 1.5 g/l bile salts (Becton Dickinson/Difco) as a selective agent. After 24 h incubation at 37°C, colony counts were performed to determine cell viability.

### Laser

Cells were irradiated at 800 nm with a 300 kHz RegA 9050 laser (Coherent, Inc.) pumped by a 10 W Verdi (Coherent, Inc.). The beam had a pulse with of ~50 fs and the rms output power attenuated to ~485±15 mW with a neutral density filter.

### Third harmonic generation

**Figure 1:**
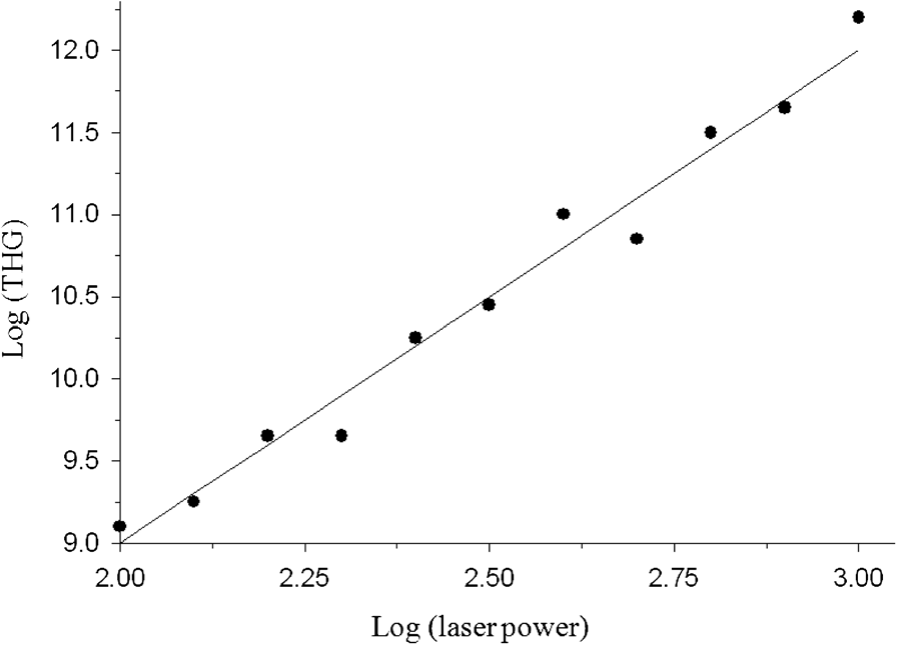
**Dependence of the log of light intensity emitted by 10 μM haemozoin in solution (in units of fW/cm**^**2**^**) as a function of the log of incident laser light intensity (in units of mW/cm**^**2**^**).** The data were fitted by a straight line with a slope of 2.81±0.3.

To test for third harmonic generation by haemozoin, light emitted by a 10 μM haemozoin solution was collected at 180° with a UV lens, passed through a bandpass filter (265±10 nm) and focused onto a PMT (Hamamatsu, Bridgewater, NJ). The intensity of the emitted light vs. incident laser intensity was plotted on a log-log graph and fitted with a straight line (Figure 1).

## Results and discussion

### Non-linear optical properties of haemozoin

The non-linear optical properties of haemozoin were studied by measuring the intensity of light emitted by haemozoin in solution as function of the intensity of the incident laser light. A log-log plot of the data were fitted with a straight line with a slope of 2.81±0.3 (Figure 1). This value is very close to the theoretical value of 3, expected from a third-power dependence and hence consistent with a third harmonic generation process (see Additional file 1 for more details).

### Laser-induced reduction in parasitaemia

**Figure 2:**
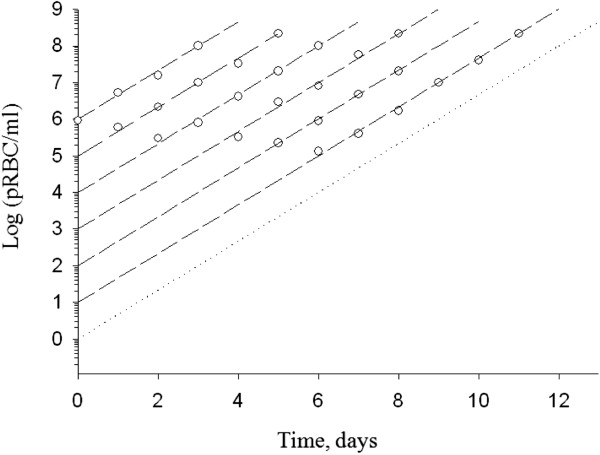
**Growth curves for *Plasmodium falciparum in vitro.*** Cultures of 10^1^ to 10^6^ parasitized RBC (pRBC) were initiated with parasites in the late-phase trophozoite phase and propagated using the continuous culture method as described in the Methods section. Experimental data points were fitted with straight-line growth curves extrapolated on the vertical axis to determine initial parasite counts. Each point represents the mean number of pRBC in triplicate cultures.

Growth curves for parasites synchronized as late-stage trophozoites were constructed by inoculating the parasite into cultures. Multiplication of parasites in culture was plotted on log-linear plots as illustrated in Figure 2 and straight-line growth curves were extrapolated on the vertical axis to determine initial parasite counts. Growth curves were parallel irrespective of inoculation doses.

**Figure 3:**
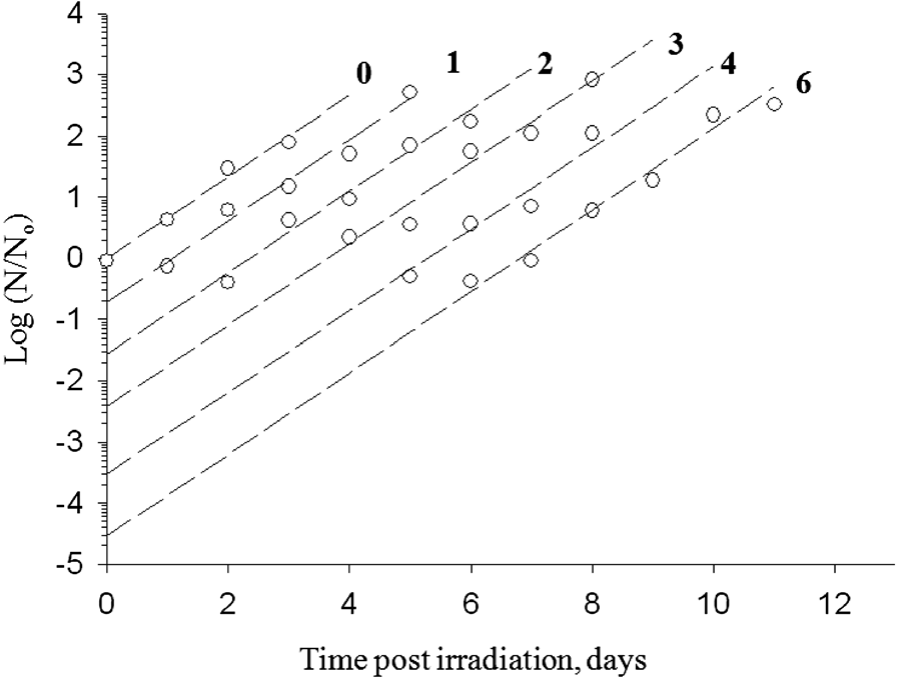
**Growth curves for late-phase trophozoite *Plasmodium falciparum *in a blood sample that was passed through a NIR laser beam for the indicated number of times and then propagated using the continuous culture method, as described in the legend to Figure **2**.** Ordinate represents the log of the number of pRBC (N) divided by the initial number of pRBC (N_o_).

The effect of irradiation on parasite viability was evaluated by plotting growth curves following various irradiation times of machine-circulated blood. Each minute the entire test volume of blood passed through the laser beam once. To determine the survival rate of parasites after irradiation with the laser, the corresponding growth curves of irradiated parasites were extrapolated on the vertical axis as shown in Figure 3.

**Figure 4:**
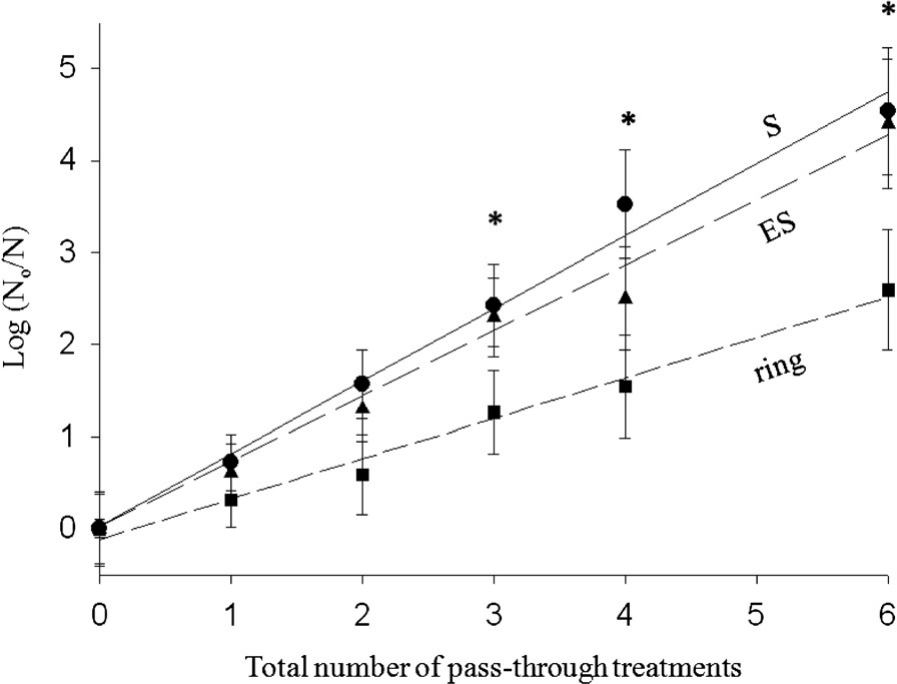
**Dose–response curves for NIR laser-induced inactivation of *Plasmodium falciparum.*** The log of ratio of number of un-irradiated parasites at the indicated phase to the number of parasites left after exposure to the laser; plotted against the number of times the entire blood sample passed through the laser beam, on a log-linear graph. Each data point represents the mean of three repetitions and the bars represent SEM. Data points with asterisks represent the number of pass-through times for which the mean value of log (N_o_/N) for ring phase parasite was statistically different (p < 0.05) from the corresponding value obtained for early and late phase parasites. ES, early trophozoites; S, late trophozoites.

Reduction in parasitaemia was quantified by fitting the ratio of remaining viable parasites to the initial un-irradiated parasite count with a single exponential decay function. This function produces a straight line on a logarithmic scale. Figure 4 plots the negative of that log ratio. As can be seen, a ~4-log reduction in parasite count for the late and early trophozoites phases were obtained following six full passes of the entire blood sample volume through the laser beam. A ~2-log reduction for the ring phase was obtained for the same irradiation regimen. The dosimetry data are summarized in Table 1.

### Bactericidal effect of NIR laser and haemozoin

**Figure 5:**
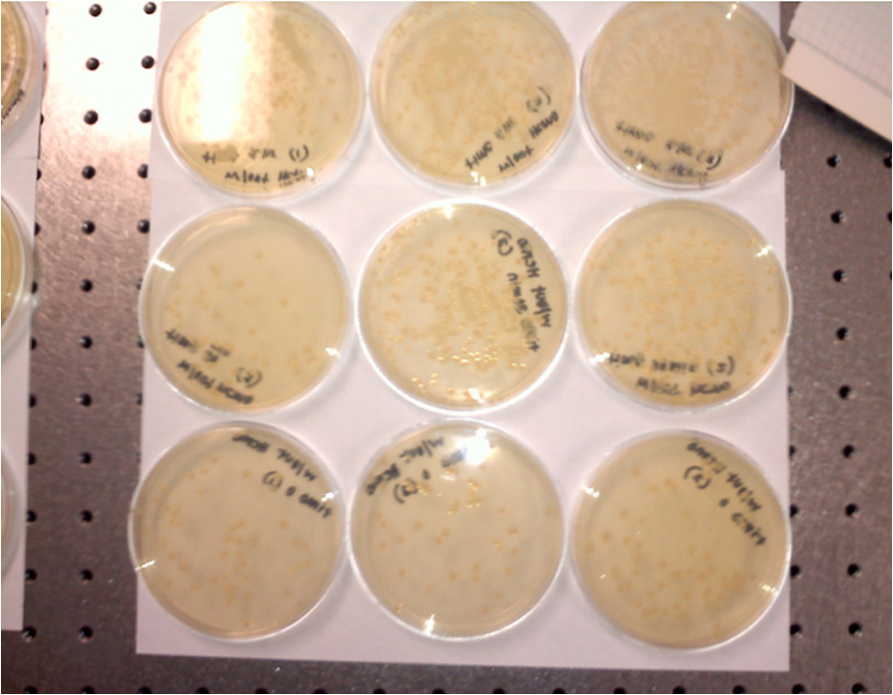
**Laser-induced bactericidal effect of haemozoin.***Escherichia coli* colonies on agar plates following 0 (*upper row*), 20 (*middle row*) and 40 (*lower row*) minutes of irradiation in the presence of 1 μM synthetic haemozoin.

**Figure 6:**
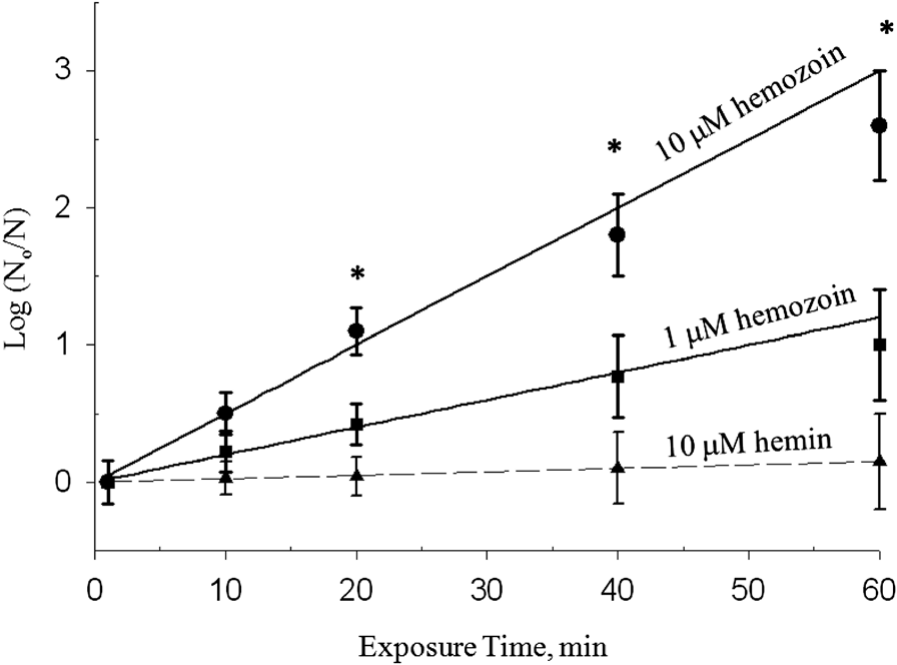
**Dose-response curves for NIR laser-induced inactivation of* E. coli *in the presence of synthetic haemozoin.** The ratio of number of bacteria left after exposure to laser to the number of un-irradiated bacteria; plotted against irradiation time on a log-linear graph. Also shown is NIR laser-induced killing of * E. coli * in the presence of 10 μM haemin. Each data point represents the mean of four repetitions and the bars represent SEM. Data points with asterisks represent exposure times for which the mean value of log (N_o_/N) in the presence of haemozoin was statistically different (p < 0.05) from the corresponding value obtained in the presence of haemin.

It is hypothesized that parasite kill in our system was caused by haemozoin-mediated UVC radiation, causing replication-defective mutations in the parasite’s DNA. To gain further insight, synthetic haemozoin crystals were added to a suspension of *E. coli* bacteria in a cuvette and the mixture irradiated with the laser under continuous stirring. Figure 5 illustrates the bactericidal effect of the pulsed NIR laser in the presence of haemozoin (1 μM) as a function of exposure time. The data for the bactericidal effect of haemozoin are plotted in Figure 6. As can be seen, a ~1-log reduction in CFU count was obtained with 1 μM haemozoin following 60 min exposure to the laser; and a ~2-log reduction with 10 μM haemozoin, for the same exposure time.

To further test the hypothesis that haemozoin’s bactericidal effect was mediated by UV radiation, due to third harmonic generation, control experiments were carried out by replacing haemozoin with haemin (a precursor of haemozoin). Haemin cannot generate UV light by THG. Illuminating the cells in the presence of haemin induced a moderate cell kill with ≤15% reduction in CFU (Figure 6**,** dashed line), possibly via a photodynamic effect [17]. The lack of a significant bactericidal effect upon treatment with haemin suggests that for the most part haemozoin in these experiments remained intact and did not revert to its precursor haemin form when put in solution.

By multiplying the concentration of haemozoin (in femtograms per parasitized RBC) obtained from cultured parasites, by the geometric mean number of parasites per microlitre in patients with mild and severe malaria, Keller *et al* estimated the blood concentration of haemozoin as ranging from 1.9 μg/ml in patients with mild symptoms to 12.9 μg/ml in patients with more severe cases of malaria [18]. These concentrations correspond to molar concentrations of 2.9 and 19.7 μM, respectively and are on the same order of magnitude as the concentrations used in our pilot bactericidal experiments.

## Conclusions

In the present work it has been shown that a pulsed NIR laser light can be used to kill *Plasmodium falciparum* in infected cultured red blood cells. A ~0.5-log reduction in parasite count has been achieved by passing the entire volume of test blood sample once through the path of a 485 mW/cm^2^ laser beam. At a normal perfusion rate of 300 ml/min it would take 20 minutes to pass the entire volume of blood of an adult male patient (~ 6 liters) once through the laser beam to achieved a ~0.5-log reduction in parasitaemia.

According to World Health Organization guidelines [19], severe malaria is defined as a case with >250,000 parasites/μl of blood; while mild malaria is defined as a case with <100,00 parasites/μl of blood. Thus, a ~0.5-log reduction in parasitaemia may be sufficient to downgrade the symptoms of malaria in a patient undergoing treatment, from severe to mild. Based on the linear log kill curves obtained in our studies (Figure 4) the kill rate can be increased linearly by adding more lasers along the dialysis perfusion line. Thus, a ~1-log reduction can be achieved in principle by passing the blood through two laser beams in tandem.

The results presented here suggest that cells that are close to schizont phase, such as the late trophozoites parasite, are more sensitive to irradiation then parasites in ring phase. However, trophozoite-infected RBCs may be sequestered within blood vessels, as a result of the up-regulation of cell surface markers that mimic endogenous cellular adhesion molecules and hence might not be fully accessible to laser treatment.

As Table 1 suggests, laser light dose required to achieve a 63% kill of *E. coli* was more than 20-fold larger than that required to kill 63% of late-phase trophozoite form of *P. falciparum* (345.2±40 J/cm^2^*vs.* 16.6±1.7 J/cm^2^). The higher sensitivity of the parasite to the treatment probably stems from the high local concentration of haemozoin within the parasite’s food vacuole and thus the closer proximity of the UVC “source” to the organism’s DNA. With that regard it should be noted that while UVC light is known to cause replication-defective mutations in bacteria and other organisms via the formation of crosslinked pyrimidine dimers [20], other cytotoxic mechanisms such as photodynamic damage may also play a role [21]. *Escherichia coli* proved to be an ideal cell model for further characterization of the kill mechanism. The genetics of *E. coli* has been studied extensively and the expression patterns of many genes in its genome well characterized [22], all of which will facilitate a thorough investigation of the kill mechanism in follow-up studies.

Maximal tissue penetration of the 800-nm laser light [13] may offer an alternative clinical approach for treating the infected patient by irradiating the blood through the skin rather than through a dialysis tubing. How would such treatment affect healthy blood cells? Light intensity decays with distance from the source following an inverse-square law. Thus assuming haemozoin within an infected RBC is an omnidirectional point source at the center of the cell and that the diameter of RBC is 8 μm, then at a distance of 16 μm from the center of the cell, the UVC intensity will fall to 1/2^2^ = 25% of the intensity at the surface; and at a distance of 24 μm to 1/3^2^ = 11% the intensity at the surface. While the inverse-square law provides for a rapid fall of the light intensity from the source, the attenuation might not be sufficient to fully protect nearby healthy endothelial, smooth muscle and other cell types. Thus, in the case of a trans-cutaneous treatment, careful light-dosimetry studies will need to be carried out to evaluate the safety of the treatment regimen.

Furthermore, it may be possible to increase the conversion efficiency of THG by rotating the haemozoin crystals for an optimal phase match between the crystal’s optical axis and the incident light beam. Magnetic fields may be utilized to rotate the crystals in the desired direction [23].

Another question with clinical implications is whether haemozoin crystals might disintegrate following interaction with the intense laser light. Damage threshold experiments of haemozoin crystal powder will need to be determined. Another concern which will need to be addressed is whether haemozoin released following parasite kill may be taken up by blood cells and induce inflammatory reactions.

Finally, computer modeling with different predator–prey models should be developed to estimate minimal light dosimetry needed to reduce parasite population in the blood to sub-critical levels.

## Abbreviations

ACT, Artemisinin-combination therapy; CFU, Colony forming units; NIR, Near infra-red; PMT, Photomultiplier tube; pRBC, Parasitized RBC; rms, Root mean square; THG, Third harmonic generation.

## Competing interests

The authors declare that they have no competing interests.

## Authors’ contributions

DL and EG carried out the experiments. RS prepared all cell media and reagents for the experiments. EG conceived the idea of using THG by haemozoin to reduce parasitaemia in malaria. EG has previously worked on the related technique of photodynamic therapy (PDT). All authors have read and approved the final version of the paper.

## Supplementary Material

Additional file 1 **Theory of third harmonic generation (THG).** Light propagating through a vacuum will obey the principle of superposition, however this is not generally true for light propagating through condensed media. Click here for file

Additional file 2 **Black-box diagram for third harmonic generation.** (top panel) A black-box diagram illustrating the principle of THG. ωo is the fundamental laser frequency. (bottom panel) Optical transitions between the different energy levels are indicated by gray arrows. The darker the shade of the arrow the larger the probability of that transition. The frequency of light emitted by THG is 3ωo. NLOC, non-linear optical crystal. Click here for file
