# Supplementary material for: Laser-induced inactivation of Plasmodium falciparum
Source: Malar J. 2012 Aug 8;11:267. doi: 10.1186/1475-2875-11-267 (PMC3464159; doi:10.1186/1475-2875-11-267)
Supplement: Additional file 1 — Theory of third harmonic generation (THG). Light propagating through a vacuum will obey the principle of superposition, however this is not generally true for light propagating through condensed media. [file 1475-2875-11-267-S1.docx]

**Additional file 1.**

Light propagating through a vacuum will obey the principle of superposition, however this is not generally true for light propagating through condensed media. As light propagates through transparent media, it induces a dipole moment on any atoms present in the propagating electromagnetic field. At sufficiently high field strengths, the induced dipole moment is no longer proportional to the applied field, thus giving rise to nonlinear optical effects.

The dipole per unit volume (called the polarization), can generally be expressed as follows;

P = *ϵ_0_*V(r)E (1)

where *ϵ_0_* is the permittivity of free-space, *V(r)* is the restoring force acting on the polarized medium as a function of electron displacement from the nucleus and *E* is the electric field within the light wave. If *V(r)* is perfectly linear, then;

P = *ϵ_0_*(1+*ϵ*)E (2)

where *ϵ* is the permittivity of the medium, and is related to the refractive index (*n*) at optical frequencies by;

*ϵ* = n^2^ (3)

At low field strengths, a linear approximation of *V(r)* is suitable and we only need characterize an optical medium by its refractive index. *V(r)* however is not linear in the general case. Nonetheless, the expression for the product *V(r)E* can be expanded as a Taylor series;

P(t) = ϰ^(1)^ E + ϰ^(2)^E^2^ + ϰ^(3)^*E^3^ +… (4)

where ϰ^(n)^ is the n-th order complex optical susceptibility of the medium. The presence of such a term is generally referred to as an *n-th* order nonlinearity. In general ϰ^(n)^ is an *n+1* order tensor representing both the polarization dependent nature of the parametric interaction as well as the symmetries (or lack thereof) of the nonlinear material. The first term on the RHS in Eqn. 4 represents linear interactions between light and matter such as one-photon absorption, reflection and refraction of light. The second term represents sum and difference frequency generation, a special case of which is optical second harmonic generation (SHG). In this case, two photons are removed from the incident light and combined to form a single photon at twice the original frequency. The third term represents a number of third-order nonlinear optical processes, including third harmonic generation. In most non-linear materials the following relationship holds ϰ^(1)^ << ϰ^(2)^ << ϰ^(3)^.

Consider now a monochromatic laser light with electric field *E(t)* and a fundamental frequency *ω_o_* applied to a non-linear material with third-order non-linear susceptibility ϰ^3^(t) :

E(t) = E cos (ω_o_t) (5)

Assuming the material possess only a non-zero ϰ^3^(t) , the polarization *P(t)* within the non-linear material consists of only the third-order non-linear polarization, *P^3^(t)*:

P^3^(t) = ϰ^3^E^3^(t) (6)

Using the identity:

cos^3^(ωt) = 0.25cos(3ωt) + 0.75cos(ωt) (7)

yields:

P^3^(t) = 0.25ϰ^3^E^3^(t) cos(3ω_o_t) + 0.75ϰ^3^E^3^(t) cos(ω_o_t) (8)

The first term on the RHS of Eqn 8 leads to generation of electromagnetic radiation at an angular frequency of 3ω_o_ by a process known as third harmonic generation. The relationship between the fundamental frequency of the laser, the non-linear crystal and third harmonic generation is schematically illustrated in Figure A1.

**Figure A1**. Third harmonic generation (THG). (*top panel*) A black-box diagram illustrating the principle of THG. ω_o_ is the fundamental laser frequency. (*bottom panel*) Optical transitions between the different energy levels are indicated by gray arrows. The darker the shade of the arrow the larger the probability of that transition. The frequency of light emitted by THG is 3ω_o_. NLOC, non-linear optical crystal.
