# Supplementary figures and images for: Laser-induced inactivation of Plasmodium falciparum
Source: Malar J. 2012 Aug 8;11:267. doi: 10.1186/1475-2875-11-267 (PMC3464159; doi:10.1186/1475-2875-11-267)

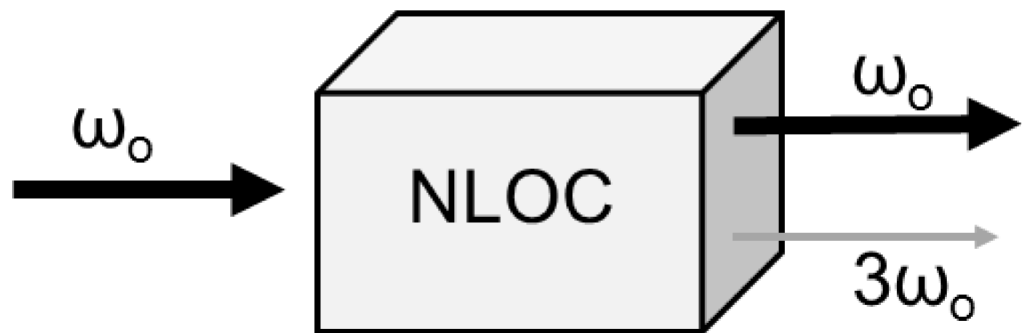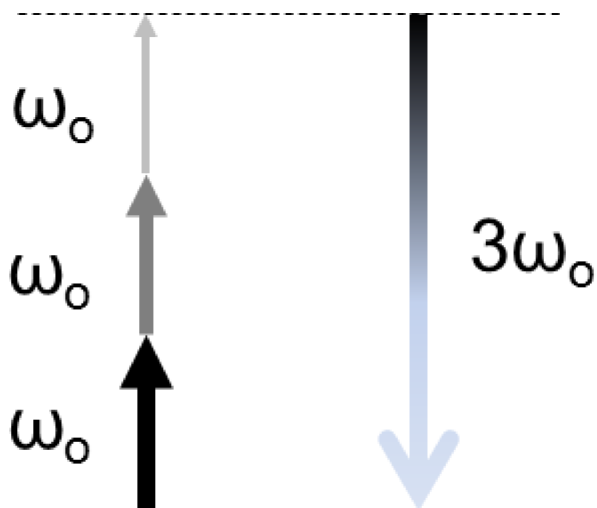

Supplement: Additional file 2 — Black-box diagram for third harmonic generation. (top panel) A black-box diagram illustrating the principle of THG. ωo is the fundamental laser frequency. (bottom panel) Optical transitions between the different energy levels are indicated by gray arrows. The darker the shade of the arrow the larger the probability of that transition. The frequency of light emitted by THG is 3ωo. NLOC, non-linear optical crystal. [file 1475-2875-11-267-S2.pdf]
